# Autoimmune Cholangitis in the SJL/J Mouse is Antigen Non-specific

**DOI:** 10.1080/1044667021000096455

**Published:** 2002-06

**Authors:** Motoko Sasaki, Jorge Allina, Joseph A. Odin, Swan N. Thung, Ross Coppel, Yasuni Nakanuma, M. Eric Gershwin

**Affiliations:** 1 Division of Rheumatology Allergy and Clinical Immunology University of California School of Medicine Davis CA 95616 USA; 2 Department of Human Pathology Kanazawa University Graduate School of Medicine Kanazawa Japan; 3 Division of Liver Diseases Mount Sinai School of Medicine New York NY 10029 USA; 4 Department of Pathology Mount Sinai School of Medicine New York NY 10029 USA; 5 Department of Microbiology Monash University Clayton Vic. 3168 Australia

## Abstract

Primary biliary cirrhosis (PBC) is an autoimmune disease characterized by intrahepatic bile duct destruction and the production of anti-mitochondrial antibodies (AMA). The absence of an animal model has been a striking impedance in defining the molecular basis of disease. Previous work has suggested that SJL/J mice immunize with the pyruvate dehydrogenase complex (PDC-E2), the major mitochondrial autoantigen of PBC, leads to the development of lymphoid cell infiltration in portal tracts and a model system coined autoimmune cholangitis. We hypothesized that this pathology would be augmented if immunization occurred in the presence of IFN-γ injections. Accordingly, SJL/J mice were immunized with PDC-E2 and, for purpose of control, α-casein. Subgroups of mice were also treated with exogenous IFN-γ. As expected, mice immunized with PDC-E2, with or without IFN-γ, developed high titer AMAs. In contrast, mice immunized with α-casein, develop antinuclear antibodies. More importantly, the livers from mice immunized with PDC-E2 and/or those immunized with α-casein all displayed lymphoid cell infiltration to the portal tracts, irrespective of bile duct size. Indeed, there was no significant difference between the experimental and the control groups by histologic analysis. Thus, autoimmune cholangitis in these mice is antigen non-specific.


*Developmental Immunology*, 2002 Vol. 9 (2), pp. 103–111

Taylor & Francis healthsciences

### Autoimmune Cholangitis in the SJL/J Mouse is Antigen Non-specific

MOTOKO SASAKI[a,b], JORGE ALLINA[c], JOSEPH A. ODIN[c], SWAN N. THUNG[d], ROSS COPPEL[e], YASUNI NAKANUMA[b] and M. ERIC GERSHWIN[a,*]

[a]*Division of Rheumatology, Allergy and Clinical Immunology, University of California School of Medicine, Davis, CA 95616, USA;* [b]*Department of Human Pathology, Kanazawa University Graduate School of Medicine, Kanazawa, Japan;* [c]*Division of Liver Diseases, Mount Sinai School of Medicine, New York, NY 10029, USA;* [d]*Department of Pathology, Mount Sinai School of Medicine, New York, NY 10029, USA;* [e]*Department of Microbiology, Monash University, Clayton, Vic. 3168, Australia*

Primary biliary cirrhosis (PBC) is an autoimmune disease characterized by intrahepatic bile duct destruction and the production of anti-mitochondrial antibodies (AMA). The absence of an animal model has been a striking impedance in defining the molecular basis of disease. Previous work has suggested that SJL/J mice immunize with the pyruvate dehydrogenase complex (PDC-E2), the major mitochondrial autoantigen of PBC, leads to the development of lymphoid cell infiltration in portal tracts and a model system coined autoimmune cholangitis. We hypothesized that this pathology would be augmented if immunization occurred in the presence of IFN-γ injections. Accordingly, SJL/J mice were immunized with PDC-E2 and, for purpose of control, α-casein. Subgroups of mice were also treated with exogenous IFN-γ. As expected, mice immunized with PDC-E2, with or without IFN-γ, developed high titer AMAs. In contrast, mice immunized with α-casein, develop antinuclear antibodies. More importantly, the livers from mice immunized with PDC-E2 and/or those immunized with α-casein all displayed lymphoid cell infiltration to the portal tracts, irrespective of bile duct size. Indeed, there was no significant difference between the experimental and the control groups by histologic analysis. Thus, autoimmune cholangitis in these mice is antigen non-specific.

*Keywords*: Anti-mitochondrial antibody; Autoimmunity; Cholangitis; Liver; Primary biliary cirrhosis; SJL/J mice

### INTRODUCTION

Primary biliary cirrhosis (PBC) is a chronic inflammatory, organ specific autoimmune disease of the liver characterized by high titer anti-mitochondrial antibodies (AMA) and intrahepatic bile duct destruction (Nakanuma and Ohta, 1979; Scheuer, 1994; Kaplan, 1996). The etiology and pathogenesis of PBC are unknown. High titer AMA react with a series of highly conserved intramitochondrial proteins including the E2 subunit of the pyruvate dehydrogenase complex (PDC-E2), the major autoantigen of PBC. Whether liver injury is caused by AMA (Malmborg *et al.*, 1998; Feehally and Allen, 1999), PDC-E2 specific T cells (Van de Water *et al.*, 1997; Shimoda *et al.*, 1998; Inada *et al.*, 2000), NK cells (Tsuneyama *et al.*, 1998; Iwata *et al.*, 2000) or pathological alterations of the bile ducts themselves (Yasoshima *et al.*, 1995; Kimura *et al.*, 1996; Leon *et al.*, 1997) are all being investigated. However, it is important to bear in mind that PBC is an autoimmune disease that most likely develops as a multi-hit disease, not just one insult. It has been theorized that PBC, in a genetically susceptible host, may be initiated by a molecular mimic (Coppel and Gershwin, 1995; Shimoda *et al.*, 2000) or a xenobiotic (Uibo and Salupere, 1999; Long *et al.*, 2001). However, to this end, no definitive causative agent has, as yet, been identified.

The first reported animal model for PBC was observed in an inbred rabbit strain with spontaneous non-suppurative cholangitis (Tison *et al.*, 1982). However, the specificity and repertoire of AMA in these rabbits failed to mimic human disease. More recently, PBC epithelial cells were injected intravenously into neonatally thymectomized A/J mice on the premise that altered epithelial cells may initiate disease (Kobashi *et al.*, 1994; Masanaga *et al.*, 1998). Also, injection of C57BL/6 mice with purified PDH in LPS or injection of recombinant polypeptides of PDC-E2 (Ide *et al.*, 1996; Kimura *et al.*, 1996) have been attempted. However, although AMAs were induced in all of these proposed

*Corresponding author. Tel.: +1-530-752-2884. Fax: +1-530-752-4669. E-mail: megershwin@ucdavis.edu

ISSN 1044-6672 print © 2002 Taylor & Francis Ltd
DOI: 10.1080/1044667021000096455

models, pathological changes in the liver similar to human PBC were not induced. In contrast, a GVHD model for PBC has also been developed which induces histological pathology but serological responses are not specific for mitochondrial antigens (Tanaka *et al.*, 1999; Zeniya, 2000). As a whole, all animal models developed to date fail to accurately mimic human PBC by the induction of both AMA and intrahepatic liver damage. With these observations in mind, Jones *et al.* (2000) reported the appearance of autoimmune cholangitis in SJL/J mice following the immunization of bovine PDC-E2, emulsified in CFA. The SJL/J mouse was chosen because it has been successfully used as models of other autoimmune disease, including experimental allergic encephalomyelitis, systemic lupus erythematosus and mercury induced immunopathology (Vidal *et al.*, 1994; Abedi-Valugerdi and Moller, 2000; Winer *et al.*, 2001). Further, T cells isolated from affected mice displayed a mixed Th1/Th2 response when stimulated with auto-antigen *in vitro* (Jones *et al.*, 1999). IFN-γ is a Th1 cytokine involved in the generation of antigen-specific T cells and has already been shown to be involved in induction of experimental myasthenia gravis (Wang *et al.*, 2000). Therefore, we hypothesized that injection of exogenous IFN-γ would enhance the development of a PBC-like disease in SJL/J mice immunized with bovine PDC-E2. However, we report herein that while SJL/J mice do demonstrate evidence of significant cholangitis, that such cholangitis is antigen non-specific regardless of IFN-γ injection and thus not reflective of PBC.

### MATERIALS AND METHODS

### Antigens

A mitochondrial fraction isolated from bovine heart tissue was prepared as described previously (Fregeau *et al.*, 1990); native PDC-E2 was thence purified (Stanley and Perham, 1980). As a negative control, α-casein, was purchased from Sigma Inc. (St Louis, MO). A lipoated, huPDC-E2 peptide (p163) spanning amino acids 163–176 (GDLLAEIETDKATI), previously shown to be a major T cell epitope (6–8), was synthesized by Alpha Diagnostic International (San Antonio, TX). Peptide purity was confirmed by reversed phase HPLC.

### Immunization

Female SJL/J mice were obtained from Jackson Laboratories (Bar Harbor, ME) and all procedures conducted according to the principles outlined in the Guidelines of the Committee on Care and Use of Laboratory Animals. In the first set of experiments, mice were immunized at 12 weeks of age with a single 200 μl intraperitoneal injection of 500 μg of either bovine PDC-E2 or α-casein emulsified in complete Freund's adjuvant as described (Jones *et al.*, 2000); the complete Freund's adjuvant (CFA) used contained 10 mg/ml *Mycobacterium tuberculosis* strain H37Ra (Difco) in incomplete Freund's adjuvant purchased from Sigma Inc. (St Louis, MO). In subgroups of mice, 3000 units of murine recombinant IFN-γ was administered intraperitoneally twice a week for four weeks (Kyuwa *et al.*, 1998). The mice were divided into the following groups: (A) PDC-E2; (B) PDC-E2 and IFNγ; (C) α-casein; and (D) α-casein and IFN-γ in groups of 8–12 animals (Table I). Mice were serially observed and by 30 weeks of age, mice were sacrificed. This time period was chosen based on earlier observations by Jones *et al.* (2000). Sera were assayed for autoantibodies. In addition, the liver, salivary gland, thymus, lung, heart, kidney, spleen, stomach, small and large intestine, were all removed for evaluation. Tissue specimens were fixed with 10% neutral buffered formalin and embedded in paraffin. Deparaffinized thin sections from each paraffin block were stained with hematoxylin and eosin for histology. Because some pilot data suggested the possibility of amyloid deposition, a Direct Fast Scarlet stain was performed and tissues viewed using polarized microscopy.

In the second set of experiments, two groups of five mice were immunized at 8 weeks of age with a single 200 ul intraperitoneal injection of the following: (E) 50 ug of p163 in incomplete Freund's adjuvant (IFA); or (F) IFA alone. Mice were serially observed and at 36 weeks of age, they were sacrificed. Sera were assayed for autoantibodies. The livers were removed for evaluation as described above.

### Autoantibodies

Antimitochondrial antibodies were assayed using a combination of ELISA, immunoblotting and immunohistochemical staining of HEp-2 cells. For ELISA, microtiter plates were coated with 10 μg/ml PDC-E2 suspended

TABLE I Serological and histological findings of SJL mice 30 weeks after immunization

| | Serological findings | | | Histological findings | | | |
|---|---|---|---|---|---|---|---|
| | Anti-PDC-E2 (%) | AMA (%) | ANA (%) | Mild BD injuries (%) | BD loss (%) | Granuloma (%) | Amyloidosis (%) |
| PDC | 100 | 100 | 0 | 100 | 0 | 100 | 100 |
| PDC+IFN-γ | 100 | 66 | 33 | 66 | 0 | 100 | 66 |
| α-Casein | 0 | 0 | 100 | 100 | 0 | 100 | 0 |
| α-Casein+IFN-γ | 0 | 0 | 75 | 75 | 0 | 100 | 0 |

BD, bile duct.

in carbonate buffer (pH 9.6) and incubated overnight at 4°C. After washing three times with 0.05% Tween-20 in PBS (PBS/Tween), plates were blocked with 3% milk in PBS for 1 h at room temperature (RT). Diluted mouse sera (1:200) was distributed into individual wells and incubated for 1 h at RT. After washing, the plates were incubated with 0.1 ml of HRP-conjugated goat anti-mouse immunoglobulin (Zymed, South San Francisco, CA) for 1 h. Reactivity was visualized by addition of 40 mM 2,2′-azinobis (3-ethylbenzthiazoline-6-sulfonic acid) dissolved in 0.05 M citric acid buffer (pH 5) containing 0.5 M $H_2O_2$. The reaction was stopped after 15 min by the addition of 5% SDS. Reactivity was measured using a Wallac spectrophotometer at a wavelength of 405 nm. Known positive and negative sera were included with each assay. For immunoblotting, samples were suspended in 250 μl of sample buffer (125 M Tris–HCl (pH6.8) containing 4% SDS, 20% glycerol and 5% 2-ME), boiled for 5 min, and resolved by SDS-PAGE using 1.5 mm-thick slab gels with a 4.75% stacking gel and a 10% separating gel. Separated proteins were transferred electrophoretically to nitrocellulose filters purchased from Micron Separations (Westboro, MA). After transfer, nitrocellulose filters were cut into strips, blocked with 3% milk in PBS for 1 h at RT and probed by incubation for 1 h with mouse sera diluted at 1:200. After washing with PBS/Tween, strips were incubated for an additional hour with HRP-labeled goat anti-mouse or anti-human polyvalent immunoglobulin (1: 2000). Strips were washed and visualized with enhanced chemiluminescent substrate purchased from Pierce (Rockford, IL). Known AMA positive PBC sera and control negative sera were used throughout these studies as controls.

Immunohistochemical staining of Hep-2 cells were performed using Hep-2 cell slides purchased from Antibodies Inc. (Davis, CA). Slides were incubated at RT for 1 h with SJL/J mice sera, human PBC sera or control sera diluted at 1:40. After incubation, slides were washed in PBS for 10 min. The reaction was followed by a 30 min incubation with FITC-conjugated goat anti-mouse or anti-human IgG diluted in PBS. Once again, known positive and negative sera were used throughout.

### RESULTS

### Induction of Antimitochondrial Antibodies

One defining serologic characteristic of PBC is the development of high titer AMA specific for the E2 subunit of PDC-E2 (Gershwin *et al.*, 2000). Mice were bled and screened for the development of AMA. All sera collected at 7 weeks from SJL/J mice immunized with PDC-E2 (group A) showed positive reactivity against PDC-E2 by immunoblot (Fig. 1, lanes 1 and 2). In contrast, sera isolated at 7 weeks from mice immunized with α-casein (group C) failed to react with PDC-E2 (Fig. 1, lane 3). All responses from mice immunized with PDC-E2 (group A)

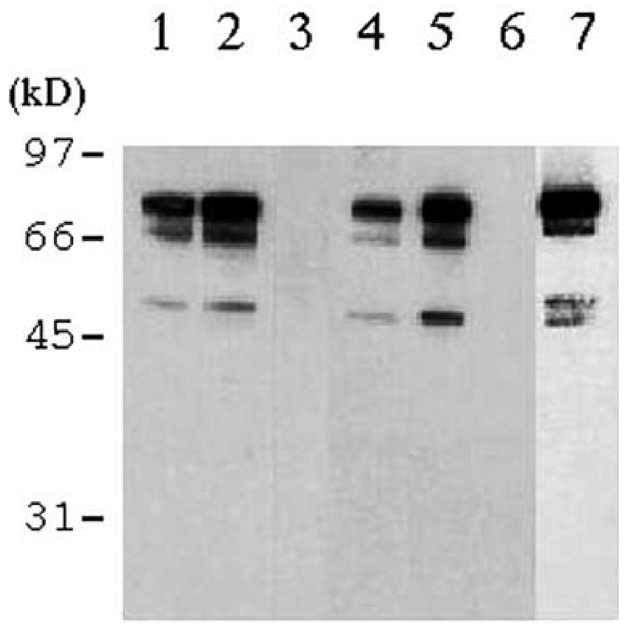

FIGURE 1 Immunization of SJL/J mice with PDC-E2 induces AMA. Sera isolated from SJL/J mice immunized with PDC-E2 as well as PBC patient sera recognized PDC-E2 (74 kD) and OGDC (48 kD). Lanes 1 and 2 represent sera isolated at 7 weeks from mice immunized with PDC-E2. Lane 3 represents sera isolated at 7 weeks from a mouse immunized with α-casein. Lanes 4 and 5 represent sera isolated at 30 weeks from mice immunized with PDC-E2. Lane 6 represents sera isolated at 30 weeks from mice immunized with α-casein. Lane 7 is serum from a PBC patient.

were similar to responses of mice immunized with PDC-E2 plus IFN-γ (group B), including reactivity with the conserved inner lipoyl domain of PDC-E2 (data not shown). At subsequent time points, through sacrifice (30 weeks), sera reactivity was very much similar to that determined at 7 weeks post-immunization (Fig. 1, lanes 4 and 5). In contrast, sera isolated at 30 weeks from mice immunized with α-casein (group C) failed to react with PDC-E2 (Fig. 1, lane 6). Results performed by immunoblot were similar to those obtained by ELISA. Further, sera from mice primed with PDC-E2 (Groups A and B) displayed a typical speckled (punctate) anti-mitochondrial staining pattern (Fig. 2a). However, a single mouse primed with PDC-E2 and who received IFN-γ (group B) demonstrated an anti-nuclear antibody pattern at a 1:40 dilution. Sera isolated from mice immunized with α-casein (groups C and D) also displayed weak anti-nuclear antibody staining patterns (Fig. 2b).

### Chronic Inflammation in Hepatic Bile Ducts

At 7 and 30 weeks post-immunization, chronic portal inflammation and granulomas were observed in all mice, irrespective of the antigen used (Table I, Fig. 3a and b). Mild bile duct injuries were prevalent as demonstrated by the irregular arrangement of the biliary epithelium (Fig. 3c). Moreover, significant lymphoid cell infiltration into the biliary epithelium and isolated apoptotic biliary

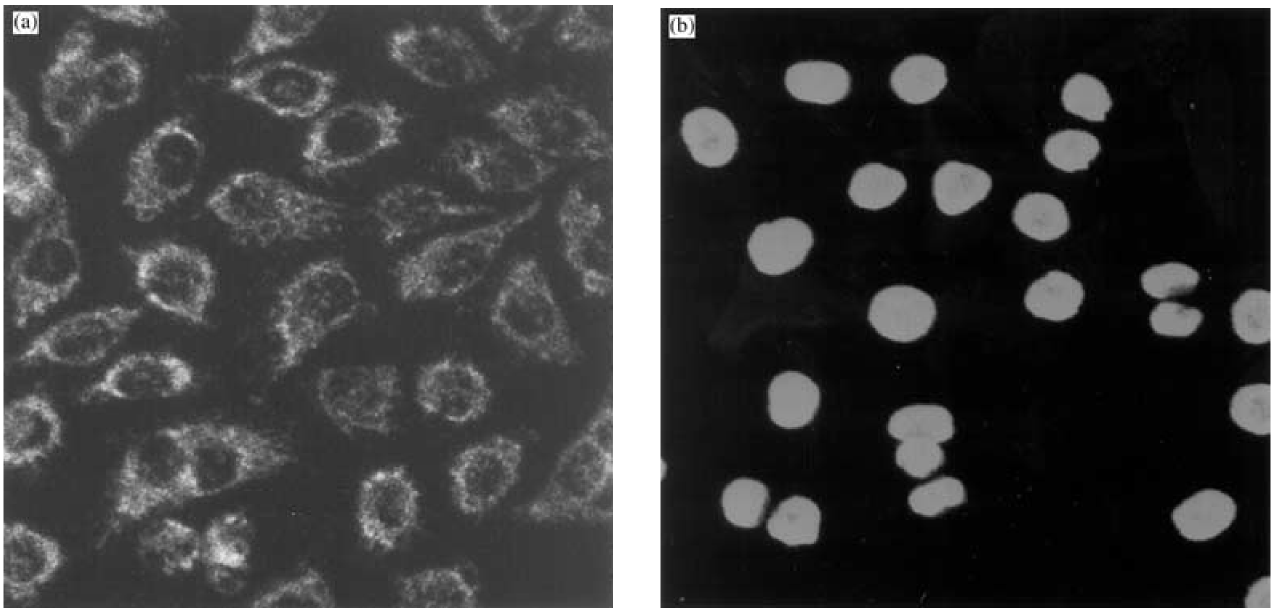

FIGURE 2 Sera isolated from SJL/J mice immunized with PDC-E2 demonstrate a speckled mitochondrial staining pattern. (a) Sera isolated from mice immunized with PDC-E2 diluted 1:40 showed a cytoplasmic speckled pattern corresponding to AMA. (b) Sera isolated from mice immunized with α-casein showed a fine staining pattern in the nucleus.

epithelial cells were observed in all groups. Of note, this pathology was observed in both small and large bile ducts (data not shown). Loss of bile ducts and destruction of the portal tracts leading to cirrhosis failed to develop in any mice.

Interestingly, focally globular amyloid depositions developed along the walls of hepatic vessels in mice immunized with bovine PDC-E2 regardless of IFN-γ treatment (Fig. 3d). These amyloid depositions were characterized as AA type as the amyloid was sensitive to $KMnO_4$ treatment. In contrast, amyloid deposition was not detected in mice injected with the α-casein and there was no significant difference in histology between α-casein groups treated with or without IFN-γ administration (data not shown).

### Selected Organs Lack Indications of Injury Due to Chronic Inflammation

Although PDC-E2 is a ubiquitous antigen found in every cell in the body, pathology is localized to small bile ducts and salivary glands. Thus, it was important to confirm the specificity of the immune response induced by injection of bovine PDC-E2 by not only analyzing liver tissue, but also selected control tissues. It was interesting that there was lymphoid cell infiltration associated with mild duct injuries in the salivary glands of mice in all groups (Fig. 3e). Moreover, after 30 weeks, lymph node swelling was evident in all mice. However, in contrast to the liver and salivary glands, there were no indications of significant inflammatory responses in other organs. Thus, immune responses to bovine PDC-E2 and α-casein with or without IFN-γ were localized to the ducts of the liver and salivary glands and appeared to be due to CFA or impurities in our antigen preparations.

### Immunization with Incomplete Freund's Adjuvant is Sufficient to Induce Portal Inflammation with Bile Duct Invasion and Damage

The initial study by Jones *et al.* (23) indicated that immunization of SJL/J mice with an immunogenic huPDC-E2 peptide (p163) was as effective as whole, bovine PDC in inducing hepatic histologic changes characteristic of PBC. To eliminate differences in antigen and CFA preparation as a reason for the difference between our results and those of Jones *et al.*, studies were conducted using synthesized p163 and IFA. At 36 weeks post-immunization, we observed significant chronic portal inflammation with bile duct invasion and damage in 2 of 5 mice injected with lipoated p163 + IFA (Table II). No granulomas were detected in any of these livers. Low titers of anti-PDC-E2 antibodies were detected by immunoblotting against purified human PDC-E2 only in the sera of the two mice with histologic changes (data not shown). Pre-incubation of sera with p163 inhibited immunoblotting of huPDC-E2. However, portal inflammation with bile duct invasion and damage, though less severe, was also observed in 3 of 5 mice injected with IFA alone. Granulomas were observed in two of these mice. Anti-PDC-E2 antibodies were not detected in the sera of these mice. Serum alkaline phosphatase levels were elevated only in the mouse (E4) with the most severe portal tract damage (data not shown). Portal inflammation with bile duct damage occurred as frequently in SJL/J mice injected with IFA alone as compared to mice injected with p163 and IFA.

(a)

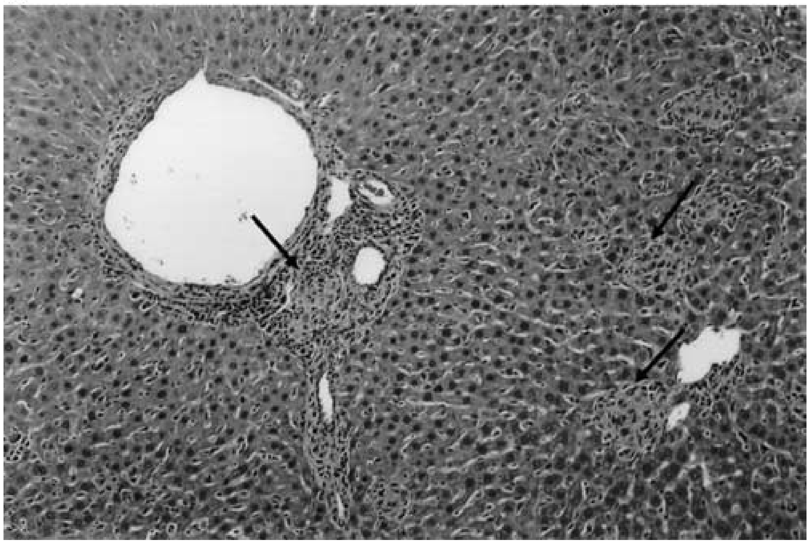

(b)

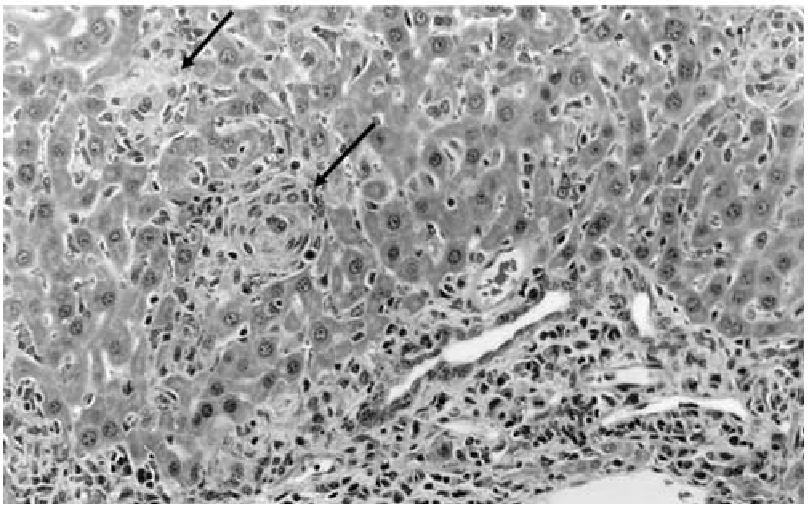

(c)

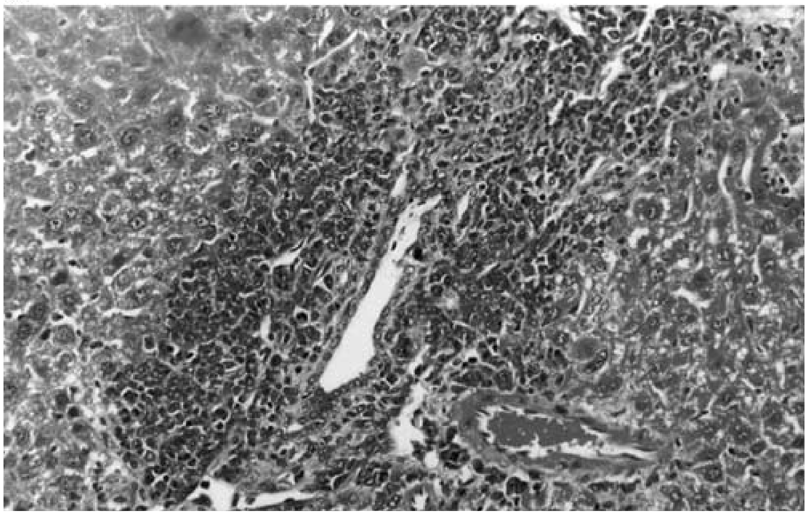

(d)

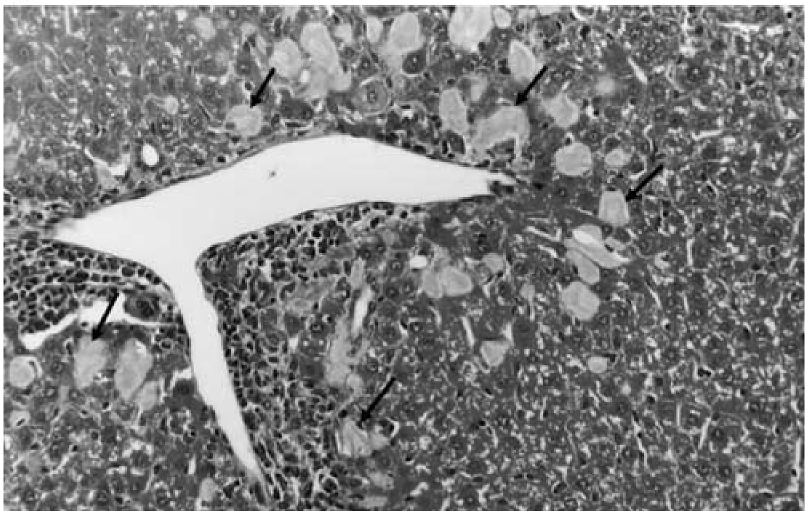

(e)

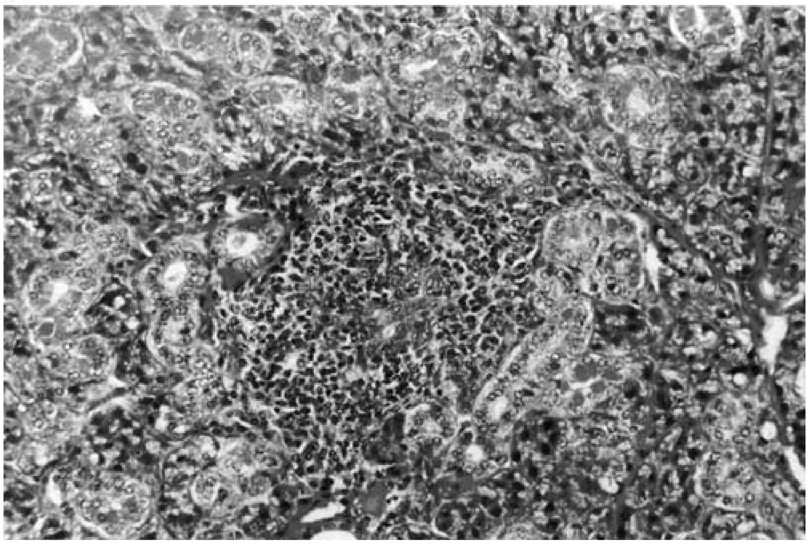

FIGURE 3 Chronic inflammation and amyloid deposition in SJL/J mice 7 and 30 weeks after immunization. (a) chronic portal infiltration and granulomas (arrows) in mice immunized with PDC-E2, 7 weeks (H&E stain, 100 × ). (b) Chronic portal infiltration, bile duct injuries and granulomas (arrows) in mice immunized with α-casein, 7 weeks (H&E stain, 200 × ). (c) Bile duct injuries and chronic portal infiltration in mice immunized with α-casein, 30 weeks (H&E stain, 200 × ). (d) Amyloid deposition in the sinusoids (arrows) of mice immunized with PDC-E2 (H&E stain, 100 × ). (e) Lymphoid cell infiltration associated with mild duct injuries in the salivary glands in mice immunized with PDC-E2 (H&E stain, 200 × ).

TABLE II Hepatic histologic changes

| | Portal inflammation | Duct invasion/damage | Granuloma | Hepatocellular inflammation |
|---|---|---|---|---|
| E1 | 0 | 0 | 0 | 1 |
| E2 | 0 | 0 | 0 | 1 |
| E3 | 3 | 2 | 0 | 1 |
| E4 | 4 | 4 | 0 | 2 |
| E5 | 0 | 0 | 0 | 1 |
| F1 | 0 | 0 | 0 | 0 |
| F2 | 1 | 1 | 1 | 1 |
| F3 | 2 | 2 | 0 | 1 |
| F4 | 1 | 1 | 1 | 2 |
| F5 | 0 | 0 | 0 | 0 |

### DISCUSSION

PBC is an autoimmune disease associated with an inflammatory response localized to the small bile duct cells of the liver (Gershwin *et al.*, 2000; Ludwig, 2000). While attempts to establish an animal model for PBC in a number of animal species, including several strains of mice, have had with little success (Krams *et al.*, 1989; Zeniya, 2000), Jones *et al.* have proposed an animal model for PBC termed "experimental autoimmune cholangitis" in which SJL/J mice are immunized with purified bovine PDC-E2 (Jones *et al.*, 2000). Both the use of SJL/J mice and bovine PDC-E2 may have been important factors in establishing this proposed model of PBC. Unclear susceptibility factors in SJL/J mice have made these mice useful for the establishment of models of other autoimmune diseases (Vidal *et al.*, 1994; Abedi-Valugerdi and Moller, 2000; Winer *et al.*, 2001). Additionally, purified bovine PDC-E2 may be more efficient at inducing an immune response for several reasons. In our hands, native PDC-E2 isolated from bovine heart tissue induces a stronger response than *E. coli*-produced recombinant protein when injected into mice (unpublished data). First, it has been suggested that lipoic acid is essential for optimal antigenicity (Fregeau *et al.*, 1990; Bassendine *et al.*, 1998; Koike *et al.*, 1998). Native protein is lipoated while synthetic peptides and recombinant proteins may not be lipoated. Second, it is important to note that bovine PDC-E2 differs from murine PDC-E2 by 28% at the amino acid level as determined by a protein blast. However, the antigenic region of bovine PDC-E2, including lipoic acid, has high homology with the murine protein and thus select regions may be recognized as a foreign antigen or altered self by the murine immune system. It thus may induce a stronger immune response against the autoantigen than a recombinant self protein or peptide (Coppel and Gershwin, 1995; Mackay, 2000).

In the study herein, we conducted a thorough serologic and histologic review to evaluate the premise that the SJL/J mouse is a suitable animal model for PBC. We also injected purified bovine PDC-E2 as the antigen to initiate the immune response in SJL/J mice. Our control group was not injected with CFA alone, as in the study by Jones *et al.*, but with α-Casein and CFA. Mild bile duct pathology was induced by immunization with bovine PDC-E2 emulsified in CFA. However, similar pathology was also noted in the mice immunized with the control protein (α-Casein) emulsified in the same adjuvant. Antigen specific AMA were successfully induced in experimental SJL/J mice but not control mice. However, the characteristic bile duct injury typical of PBC failed to develop even after 30 weeks, although mild bile duct pathology and portal inflammation persisted. Furthermore, inflammation and diffuse bile duct pathology were observed, irrespective of bile duct size. In contrast, only small bile ducts are damaged in human PBC and affected bile ducts are distributed sparsely, not diffusely (Sasaki *et al.*, 2000).

To magnify the inflammatory environment in SJL/J mice, we injected exogenous IFN-γ but such injections failed to alter the pathology of PBC. The addition of IFN-γ did not change the specificity or intensity of histologic or serologic effects. These results are not surprising since the pathology observed herein is antigen non-specific. Hence, this system is different from the disease associated features of IFN-γ when NOD mice are injected with exogenous cytokines (Xiang and Zaccone, 1999). Alternatively, treatment of IFN-γ may cause class-switching of antigen specific AMA to IgG1. It is possible that IgG1 is not pathogenic. In fact, recently our lab has demonstrated that IgA may significantly contribute to the pathology of PBC (Reynoso-Paz *et al.*, 2000). In this respect, we should note that mice do not have IgA receptors on their bile duct cells and, in fact, have a significantly different mucosal immune system than that of humans (Gershwin *et al.*, 2000).

To eliminate non-specific effects caused by impurities in our antigen or adjuvant preparations, SJL/J mice were also injected with a lipoated, immunogenic huPDC-E2 peptide (aa 163–176) emulsified in IFA or with IFA alone. Portal inflammation with bile duct invasion and damage occurred with similar frequency in both groups. There is no indication that the development of portal inflammation was specific to a loss of tolerance to PDC-E2, though the severity of inflammation was greater in mice expressing anti-PDC-E2 autoantibodies. Thus, both the development of autoantibodies and portal tract inflammation may simply reflect the severity of the inflammatory response following ip injection. SJL/J mice appear to be predisposed to the development of portal inflammation with minimal provocation.

It is of interest that systemic amyloidosis of AA type was induced in most of the SJL/J mice immunized with PDC-E2 but not in controls with α-casein. Generally, AA type amyloidosis occurs secondary to systemic chronic inflammatory changes such as rheumatoid arthritis and tuberculosis. Heretofore, AA amyloidosis in mice has been previously induced in a number of strains of mice by repeated injections of α-casein, chemicals and endotoxin (Skinner *et al.*, 1977; Baumal, 1979; Rokita *et al.*, 1989; Kisilevsky and Young, 1994; Sipe, 1994), and in a transgenic mouse model carrying human IL-6 gene with mouse metallothionein-I promoter (Solomon *et al.*, 1999). Spontaneous amyloid deposits have also been noted in aged SJL/J mice. In fact, the injection of amyloid enhancing factor (AEF) together with α-casein in CFA induced AA amyloidosis in SJL/J mice, while α-casein alone did not induce amyloidosis (Rokita *et al.*, 1989). Our findings are in agreement with these findings but not those of Jones *et al.* (1999).

The SJL/J mouse is an interesting model for a number of diseases and it remains possible that with further study, significant information may be forthcoming on the inflammatory environment within the liver and salivary glands. However, it appears that such responses are antigen non-specific.

### *References*

Abedi-Valugerdi, M. and Moller, G. (2000) "Contribution of H-2 and non-H-2 genes in the control of mercury-induced autoimmunity", *Int. Immunol.* **12**, 1425–1430.

Bassendine, M., Palmer, J., Decruz, D., McCaugham, G., Strickland, D., Sedgewick, J. and Yeaman, S. (1998) "Approaches to a murine model of AMA positive non-suppurative destructive cholangitis (NSDC)", *J. Hepatol.*, 28, Abstract.

Baumal, R. (1979) "Similarity of casein- and endotoxin-induced, myeloma-associated and aged SJL-J amyloid in various strains of mice", *Int. Arch. Allergy Appl. Immunol.* **59**, 20–27.

Coppel, R. and Gershwin, M. (1995) "Primary biliary cirrhosis: the molecule and the mimic", *Immunol. Rev.* **144**, 17–49.

Feehally, J. and Allen, A. (1999) "Pathogenesis of IgA nephropathy", *Ann. Med. Intern. (Paris)* **150**, 91–98.

Fregeau, D., Prindiville, T., Coppel, R., Kaplan, M., Dickson, E. and Gershwin, M. (1990) "Inhibition of alpha-ketoglutarate dehydrogenase activity by a distinct population of autoantibodies recognizing dihydrolipoamide succinyltransferase in primary biliary cirrhosis", *Hepatology* **11**, 975–981.

Gershwin, M., Ansari, A., Mackay, I., Nakanuma, Y., Nishio, A., Rowley, M. and Coppel, R. (2000) "Primary biliary cirrhosis: an orchestrated immune response against epithelial cells", *Immunol. Rev.* **174**, 210–225.

Ide, T., Sata, M., Suzuki, H., Uchimura, Y., Murashima, S., Shirachi, M. and Tanikawa, K. (1996) "An experimental animal model of primary biliary cirrhosis induced by lipopolysaccharide and pyruvate dehydrogenase", *Kurume Med. J.* **43**, 185–188.

Inada, H., Yoshizawa, K., Ota, M., Katsuyama, Y., Ichijo, T., Umemura, T., Tanaka, E. and Kiyosawa, K. (2000) "T cell repertoire in the liver of patients with primary biliary cirrhosis", *Hum. Immunol.* **61**, 675–683.

Iwata, M., Harada, K., Hiramatsu, K., Tsuneyama, K., Kaneko, S., Kobayashi, K. and Nakanuma, Y. (2000) "Fas ligand expressing mononuclear cells around intrahepatic bile ducts co-express CD68 in primary biliary cirrhosis", *Liver* **20**, 129–135.

Jones, D., Palmer, J., Yeaman, S., Kirby, J. and Bassendine, M. (1999) "Breakdown of tolerance to pyruvate dehydrogenase complex in experimental autoimmune cholangitis: a mouse model of primary biliary cirrhosis", *Hepatology* **30**, 65–70.

Jones, D., Palmer, J., Kirby, J., De Cruz, D., McCaughan, G., Sedgwick, J., Yeaman, S., Burt, A. and Bassendine, M. (2000) "Experimental autoimmune cholangitis: a mouse model of immune-mediated cholangiopathy", *Liver* **20**, 351–356.

Kaplan, M. (1996) "Primary biliary cirrhosis", *N. Engl. J. Med.* **335**, 1570–1580.

Kimura, T., Suzuki, K., Inada, S., Hayashi, A., Isobe, M., Matsuzaki, Y., Tanaka, N., Osuga, T. and Fujiwara, M. (1996) "Monoclonal antibody against lymphocyte function-associated antigen 1 inhibits the formation of primary biliary cirrhosis-like lesions induced by murine graft-versus-host reaction", *Hepatology* **24**, 888–894.

Kisilevsky, R. and Young, I. (1994) "Pathogenesis of Amyloidosis", In: Husby, G., ed, *Bailliere's Clinical Rheumatology* (Bailliere indal, London) **Vol. 8**, pp 613–626.

Kobashi, H., Yamamoto, K., Yoshioka, T., Tomita, M. and Tsuji, T. (1994) "Nonsuppurative cholangitis is induced in neonatally thymectomized mice: a possible animal model for primary biliary cirrhosis", *Hepatology* **19**, 1124–1430.

Koike, K., Ishibashi, H. and Koike, M. (1998) "Immunoreactivity of porcine heart dihydrolipoamide acetyl- and succinyl-transferases (PDC-E2,OGDC-E2) with promary biliary cirrhosis sera: characterization of the autoantigenic region and effects of enzymic delipoylation and relipoylation", *Hepatology* **27**.

Krams, S., Surh, C., RL, C., Ansari, A., Reubner, B. and Gershwin, M. (1989) "Immunization of experimental animals with dihydrolipoamide acetyltransferase, as a purified recombinant polypeptide, generates mitochondorial antibodies but not primary biliary cirrhosis", *Hepatology* **9**, 411–416.

Kyuwa, S., Tagawa, Y., Shibata, S., Doi, K., Machii, K. and Iwakura, Y. (1998) "Murine coronavirus-induced subacute fatal peritonitis in C57BL/6 mice deficient in gamma interferon", *J. Virol.* **72**, 9286–9290.

Leon, M., Bassendine, M., Gibbs, P., Thick, M. and Kirby, J. (1997) "Immunogenicity of biliary epithelium: study of the adhesive interaction with lymphocytes", *Gastroenterology* **112**, 968–977.

Long, S., Quan, C., Van de Water, J., Nantz, M., Kurth, M., Barsky, D., Colvin, M., Lam, K., Coppel, R., Ansari, A. and Gershwin, M. (2001) "Immunoreactivity of organic mimeotopes of the E2 component of pyruvate dehydrogenase: connecting xenobiotics with primary biliary cirrhosis", *J. Immunol.* **167**, 2956–2963.

Ludwig, J. (2000) "The pathology of primary biliary cirrhosis and autoimmune cholangitis", *Baillieres Best Pract. Res. Clin. Gastroenterol.* **14**, 601–613.

Mackay, I. (2000) "Autoimmunity and primary biliary cirrhosis", *Baillieres Best Pract. Res. Clin. Gastroenterol.* **14**, 519–533.

Malmborg, A., Shultz, D., Luton, F., Mostov, K., Richly, E., Leung, P., Benson, G., Ansari, A., Coppel, R., Gershwin, M. and Van de Water, J. (1998) "Penetration and co-localization in MDCK cell mitochondria of IgA derived from patients with primary biliary cirrhosis", *J. Autoimmun.* **11**, 573–580.

Masanaga, T., Watanabe, Y., Van De Water, J., Leung, P., Nakanishi, T., kajiyama, G., Reubner, B., Coppel, R. and Gershwin, M. (1998) "Induction and persistence of immune-mediated cholangiohepatitis in neonatally thymectomized mice", *Clin. Immunol. Immunopathol.* **89**, 141–149.

Nakanuma, Y. and Ohta, G. (1979) "Histometric and serial section observations of the intrahepatic bile ducts in primary biliary cirrhosis", *Gastroenterology* **76**, 1326–1332.

Reynoso-Paz, S., Leung, P., Van De Water, J., Tanaka, A., Munoz, S., Bass, N., Lindor, K., Donald, P., Coppel, R., Ansari, A. and Gershwin, M. (2000) "Evidence for a locally driven mucosal response and the presence of mitochondrial antigens in saliva in primary biliary cirrhosis", *Hepatology* **31**, 24–29.

Rokita, H., Shirahama, T., Cohen, A. and Sipe, J. (1989) "Serum amyloid A gene expression and AA amyloid formation in A/J and SJL/J mice", *Br. J. Exp. Pathol.* **70**, 327–335.

Sasaki, M., Ansari, A., Nakanuma, Y., Coppel, R., Keeffe, E. and Gershwin, M. (2000) "The immunopathology of primary biliary cirrhosis: thoughts for the millennium", *Arch. Immunol. Ther. Exp.* **48**, 1–10, Warsz.

Scheuer, P. (1994) *Liver Biopsy Interpretation*, Ed. 5 (Saunders, London).

Shimoda, S., Van de Water, J., Ansari, A., Nakamura, M., Ishibashi, H., Coppel, R., Lake, J., Keeffe, E., Roche, T. and Gershwin, M. (1998) "Identification and precursor frequency analysis of a common T cell epitope motif in mitochondrial autoantigens in primary biliary cirrhosis", *J. Clin. Invest.* **102**, 1831–1840.

Shimoda, S., Nakamura, M., Shigematsu, H., Tanimoto, H., Gushima, T., Gershwin, M. and Ishibashi, H. (2000) "Mimicry peptides of human PDC-E2 163–176 peptide, the immunodominant T-cell epitope of primary biliary cirrhosis", *Hepatology* **31**, 1212–1216.

Sipe, J. (1994) "Amyloidosis", *Crit. Rev. Clin. Lab. Sci.* **31**, 325–354.

Skinner, M., Shirahama, T., Benson, M. and Cohen, A. (1977) "Murine amyloid protein AA in casein-induced experimental amyloidosis", *Lab. Invest.* **36**, 420–427.

Solomon, A., Weiss, D., Schell, M., Hrncic, R., Murphhy, C., Wall, J., McGavis, D., Pan, H., Kabalka, G. and Paulus, M. (1999) "Transgenic mouse model of AA amyloidosis", *Am. J. Pathol.* **154**, 1267–1272.

Stanley, J. and Perham, R. (1980) "Purification of the 2-oxo acid dehydrogenase complexes from ox heart by a new method", *Biochem. J.*, 147–154.

Tanaka, A., Lindor, K., Gish, R., Batts, K., Shiratori, Y., Omata, M., Nelson, J., Ansari, A., Coppel, R., Newsome, M. and Gershwin, M. (1999) "Fetal microchimerism alone does not contribute to the induction of primary biliary cirrhosis", *Hepatology* **30**, 833–838.

Tison, V., Callea, F., Morisi, C., Mancini, A. and Desmet, V. (1982) "Spontaneous 'primary biliary cirrhosis' in rabbits", *Liver* **2**, 152–161.

Tsuneyama, K., Yasoshima, M., Harada, K., Hiramatsu, K., Gershwin, M.E. and Nakanuma, Y. (1998) "Increased CD1d expression on small bile duct epithelium and epithelioid granuloma in livers in primary biliary cirrhosis", *Hepatology* **28**, 620–623.

Uibo, R. and Salupere, V. (1999) "The epidemiology of primary biliary cirrhosis: immunological problems", *Hepatogastroenterology* **46**, 3048–3052.

Van de Water, J., Shimoda, S., Niho, Y., Coppel, R., Ansari, A. and Gershwin, M. (1997) "The role of T cells in primary biliary cirrhosis", *Semin. Liver Dis.* **17**, 105–113.

Vidal, S., Gelpi, C. and Rodriguez-Sanchez, J. (1994) "(SWR × SJL)F1 mice: a new model of lupus-like disease", *J. Exp. Med.* **179**, 1429–1435.

Wang, H., Shi, F., Li, H., van der Meide, P., Ljunggren, H. and Link, H. (2000) "Role for interferon-gamma in rat strains with different susceptibility to experimental autoimmune myasthenia gravis", *Clin. Immunol.* **95**, 156–162.

Winer, S., Astsaturov, I., Cheung, R., Schrade, K., Gunaratnam, L., Wood, D., Moscarello, M., O'Connor, P., McKerlie, C., Becker, D. and Dosch, H. (2001) "T cells of multiple sclerosis patients target a common environmental peptide that causes encephalitis in mice", *J. Immunol.* **166**, 4751–4756.

Xiang, M., Zaccone, P., Di Marco, R., Harris, R., Magro, G., Di Mauro, M., Meroni, P., Garotta, G. and Nicoletti, F. (1999) "Failure of exogenously administered interferon-gamma or blockage of endogenous interleukin-4 with specific inhibitors to augment the incidence of autoimmune diabetes in male NOD mice", *Autoimmunity* **30**, 71–80.

Yasoshima, M., Nakanuma, Y., Tsuneyama, K., Van de Water, J. and Gershwin, M. (1995) "Immunohistochemical analysis of adhesion molecules in the micro-environment of portal tracts in relation to aberrant expression of PDC-E2 and HLA-DR on the bile ducts in primary biliary cirrhosis", *J. Pathol.* **175**, 319–325.

Zeniya, M. (2000) "Lessons from animal models of primary biliary cirrhosis", *J. Gastroenterol. Hepatol.* **15**, 342–343.

